# Researches upon Some Questions Relative to Septicæmia

**Published:** 1873-02

**Authors:** H. M. Davaine

**Affiliations:** Paris


### Translation.

ARTICLE V.—*Researches upon some Questions Relative to Septicæmia.* By H. M. DAVAINE, of Paris. Translated from the French by Prof. J. W. FREER, M.D., Chicago.

"The confusion that has been made between maladies caused by 'charbon,' or malignant pustule, and those determined by the introduction into the animal economy of putrid matters, has existed until the last few years. The study of charbon has caused me to search for characters of differentiation among affections caused by putrefaction, and consequently to the investigation of septicæmia.

"Wishing to place myself under the simplest conditions, I obtained healthy beef's blood from the slaughter-house, and guarded it until it was putrefied, when I inoculated it in divers animals, but as I will show in one of the communications that I hope to have the honor of making to the Academy upon this subject, the conditions of putrefied blood are more complex than one would have anticipated, and it was only after repeated researches, that I was able to obtain, upon certain points, results that were free from doubt.

"On the 1st of February, 1869, I communicated to the Academy of Sciences some of these results, nevertheless I announced that my labors on this subject were still incomplete.

"One of the points in my communication related to the contagiousness of septicæmia; I then recalled the experiments of M. Raimbert, who reported in 1859, the transmission in many animals successively, of a malady determined by putrefied matter that had been inoculated in the first of the series, nevertheless, believing that the result was of the nature of charbon, our learned confrere did not sufficiently recognize the importance of his observations.

"M. M. Coze and Feltz, have discovered another important fact, 'which is, that in creating several generations of infected blood, by inoculation, the last of the series are toxicologically more active than the first, or even the putrid matter itself, that is, it requires more time to kill an animal with putrid matter, than by inoculation with blood of an infected animal. This experimental fact is of the greatest importance; it makes us comprehend how an epidemic is aggravated by successive transmissions.'

"I should say, in order to render homage to truth, and to one of our most illustrious masters, that Magendie recognized the contagious transmission of septicæmia; he has even stated that the virulence of the blood of an inoculated animal is greater than the original putrid matter.

"It has appeared to me that if blood, putrefied in open air, is more or less active than that of an animal killed by inoculation of this fluid, one should be able to produce precise phenomena—death for example—with different doses of these two fluids."

The following is the method pursued by M. Davaine in his experiments:

"I inject with a Pravaz syringe in the subcutaneous tissue, a determined quantity of the liquid. When it concerns fractions of drops, I mix a drop of septic blood with ten, twenty, or a hundred of water, and inject a single drop of the mixture. Quantities more infinitesimal are obtained by successive dilutions.

"Let us see at first what is the quantity of putrefied blood required to kill an animal. In order to resolve this question, it will suffice to give the abstract of the inoculations which I have practiced for divers researches during many years, nevertheless, I will only speak of those performed upon rabbits and *cobayas.*

"Of seventy-two cobayas, injected with one to ten drops of putrefied blood, forty-three survived, and twenty-five died. Of eleven other cobayas that received a fraction of a drop, none died with a dose of less than one-fourth of a drop.

"Of forty-eight rabbits, inoculated in the same manner with one to six drops, twenty-two survived, and twenty-six died. Of nine others injected with fractions of a drop, none died with a dose less than two-thousandths of a drop.

"From the above we may conclude that the introduction of one or more drops of putrefied blood into the circulation of the aforesaid animals, is not mortal in half of the cases.

"Concerning fractional drops, the cobaya succumbs rarely to less than one-tenth and the rabbit to less than one-hundredth. The extreme seems to be one-fortieth for the first, and one-two thousandth for the second.

"Observe now the second question we have to resolve, viz.: What dose of septicæmic blood—that is to say, blood taken from an animal that has died from inoculation with putrefied blood—is required to produce death in another animal of the same species?

"It would take too long to quote all of my experiences upon this subject; I will limit myself by passing in review a series of twenty-five successive inoculations, or twenty-five generations, which will give a sufficiently precise response.

"The blood of a beef taken ten days after slaughter, in July, and very much putrefied, was injected in the subcutaneous tissue of the necks of five rabbits, in doses of two, four, ten, twelve and fifteen drops. All died; the first in five days, the second in nine days, the third in forty hours, the fourth in twenty-six days, and the fifth in fifty-five days, after inoculation.

"I will have to speak, further on, of the prolonged duration of life of four of the animals, and the irregularity of this duration relatively to the quantity of liquid received.

"The blood from the heart of the rabbit that died forty hours after injection with ten drops, was injected twelve hours after in the cellular tissue of four other rabbits. The four having received successively, one, two, three and four drops, died the same night—thirty to forty hours after inoculation.

"In order not to abuse the time accorded me by the Academy, I pass to the fifth generation of inoculations: the blood from the heart of a rabbit of the fourth generation, was injected, two hours and a half after death, in three others, in doses of one, one-tenth, and one-hundredth of a drop. Two died in fourteen hours, and the third in twenty hours.

"I pass to the tenth generation: Three rabbits were inoculated with blood of the ninth generation, dead since one hour. One received one drop, another one-ten thousandth, and the third one-twenty thousandth. The first died the following night, the second fifteen hours, and the third thirty-five hours after injection.

"From the fifteenth generation, three rabbits were inoculated with one-twenty thousandth, one-thirty thousandth and one-forty thousandth of a drop. All died in intervals of from twenty to forty hours.

"The blood from a rabbit of the twentieth generation, one hour after death, was injected in doses of $\frac{500}{1000}$, $\frac{1}{100000}$ and $\frac{100}{100000}$ of a drop; of the three that had received these minimum quantities of blood, the first and third died in thirty-five hours, the second in twenty-one hours.

"In the generations following, I arrived at quantities, the minuteness of which exceeded all prevision.

"From the twenty-second generation, three rabbits were inoculated with $\frac{1}{100000}$, $\frac{1}{1000000000}$ and $\frac{1}{10000000000}$ of a drop and another with $\frac{10}{10000000000}$ of a drop of the blood of a rabbit dead since two hours, having been inoculated itself with $\frac{500}{1000000}$ of a drop of septicæmic blood. The three died at intervals of from thirty-six to forty hours.

"From the twenty-third generation, a rabbit was inoculated with $\frac{100}{10000000}$ of a drop, another with $\frac{10}{10000000000}$ of a drop. Both died in about thirty-six hours after.

"From the twenty-fourth generation, five rabbits were inoculated with the blood of one that had died from the effects of $\frac{100}{10000000}$ of a drop.

"The first received $\frac{100}{10000000}$, the second $\frac{1}{10000000000}$, the third $\frac{10}{100000000000}$, the fourth $\frac{100}{10000000000}$, and the fifth $\frac{1}{10000000000000}$ of a drop of blood from the rabbit mentioned. All of these animals died in less than twenty-four hours.

"Finally: from the twenty-fifth generation, four rabbits received $\frac{1}{10000000000000}$, $\frac{100}{10000000000000}$ and $\frac{1}{10000000000000000}$ of a drop from a rabbit of the above-mentioned series that had died with $\frac{1}{10000000000000}$ of a drop; but only one of these animals died. which was the one that had received $\frac{10}{10000000000000}$ of a drop of blood.

"It seems then that the limit of transmissibility of septicæmia with the rabbit may be $\frac{1}{1000000000000}$ of a drop of septic blood.

"In order to achieve the elucidation of the subject that I have proposed to resolve, it remains to recall the results of inoculation with blood putrefied in open air, and those inoculated with blood from animals that had died with septicæmia. We know, on the one hand, that more than one-half survived the effects of putrefied blood, while on the other, all died from infinitesimal doses of septicæmic blood. Septicæmic virus, therefore, acquires a much greater activity in passing through the blood of a living animal.

"There is another difference between blood that is putrid, or that which is septicæmic, which merits consideration, a difference already signalized by M. M. Coze and Feltz; which is the relative rapidity of death in the two cases.

"I limit myself by simply recalling the results of inoculation with the putrefied blood, which has been the source of the series of which I have made the exposition, viz.: of the five rabbits inoculated with putrefied blood, the duration of life has been from thirty to forty hours with two, and six, nine and twenty-six days with the others; while sixty-nine rabbits inoculated from the generations that followed, with the exception of two, all succumbed in less than forty hours.

"A fact worthy of notice is the irregularity of the duration of life after inoculation. The incubation or duration of the malady is not in relation with the dose administered, as we have seen with the five rabbits inoculated with the putrefied blood, of which the third with ten drops died in forty hours, and the fourth with twelve drops died in twenty-six hours. The same irregularity appeared with those that died of septicæmia. I mention but the last two of the series of which we have spoken, viz.: one with $\frac{1}{1000000000000}$ of a drop, died in twenty-two hours, the other with a stronger dose lived thirty-five hours.

"With the maladies attending 'malignant pustule,' on the contrary, one remarks a great regularity. The duration of life after injection with the infectious blood is proportional to the dose inoculated.

"We know that the virus from charbon destroys itself by putrefaction; now in place of inoculating with fractions of a drop of fresh blood, if one takes that which is putrefied, by conservation more or less protracted, the length of life is prolonged in ratio to the length of time that the virus has been subjected to putrefying influences.

"The facts that I come to notice, may throw some light upon a question incompletely understood, or controverted, relative to the pernicious effects of putrefied animal substances.

"Although at the present time we may generally be in accord upon this subject, yet not long since, the dangers arising from these emanations were denied by excellent observers; such as Parent, Duchatelet, etc. On the other hand, people in the country attribute grave maladies, such as charbon, to infection from flies that have come from putrefying cadavers. Physicians recognize the redoubtable accidents, determined by dissecting wounds, and we know that cadavers of puerperal women are particularly infectious, nevertheless, it is often impossible to explain the variability in the gravity of the accidents, which has been usually placed to the account of individual predisposition.

"Doctor Calles has remarked that fresh cadavers are more dangerous than those which have arrived at a certain stage of putrefaction. This observation appears strange, but will find its explication in the facts that I now report.

"We have already learned that blood putrefied in the open air is rarely fatal in doses of less than one drop, and sometimes it requires ten or fifteen to cause death, whilst the action of blood from an animal that has succumbed from inoculation is infectious in infinitesimal quantities; this without doubt is the cause of the virulence or non-virulence of certain cadavers.

"I will explain by an example: Two horses are wounded on the field of battle; one dies in a few hours, the other survives for a longer period; but the flesh is lacerated, blood is extravasated under the skin, in the connective tissue and elsewhere. The weather is warm and moist; in less than twenty-four hours the blood putrefies and the horse dies septicæmic soon after.

"Thus, the cadaver of the second horse, and upon the same field with the first, and whose death seemed to be due to the same original cause, is extremely infectious, while that of the first is hardly inoculable. Inoculation with the liquid from one or the other will result very differently, and if the flies that make their repast upon the blood of these two animals, alight upon the wounds of other animals, their contact remains inoffensive in one case, and in the other produces the gravest accidents.

"In order to confirm these views I made the following experiments: A meat fly being placed under a bell-glass, I introduced beneath a little blood from a rabbit that had died the evening before from septicæmia, caused by inoculation with $\frac{1}{100000000}$ of a drop of septicæmic blood. One-half hour after, I separated the sucker of this fly with scissors, and introduced it through a very narrow puncture, under the skin, behind the ear of a vigorous rabbit. It died thirty-five hours after.

"A natural question to present, is that of the duration, longer or shorter, of the virulence of septicæmia, in a certain number of generations. Does the virus diminish in power? Does it exhaust itself at last? Or, on the contrary, does it augment in activity by successive transmissions?

"The solution of these questions is not without interest in point of view of the origin, or of the intensity of epidemic and contagious diseases; for although each of these maladies may have its own proper nature or species, the knowledge of particular facts may have at least the advantage of rectifying misconceptions.

"The question of the increase of virulence by successive generations is proved by the following experiments:

"First. Beef's blood preserved ten days, was inoculated in five rabbits in doses of $\frac{1}{10}$, $\frac{1}{50}$, $\frac{1}{100}$, $\frac{1}{500}$, and $\frac{1}{1000}$ of a drop. The last two were not sick, at least not in appearance; the first three died. The limit of septicite of putrefied blood, capable of destroying life, is therefore inferior to five-hundredths of a drop.

"Second generation. The blood from the heart of the rabbit that had died from the effects of one-tenth of a drop was inoculated in five rabbits, in doses of $\frac{10}{1000}$, $\frac{20}{1000}$, $\frac{30}{1000}$, $\frac{40}{1000}$ and $\frac{50}{1000}$ of a drop. All died, in intervals of from thirty to sixty hours.

"Second experiment — First generation. Beef's blood preserved five days, was inoculated in five rabbits, in doses of one drop, $\frac{1}{100}$, $\frac{1}{1000}$, $\frac{2}{1000}$, and $\frac{1}{10000}$ of a drop. Only the first three died. The power of the virus to kill in these cases was below two-thousandths of a drop.

"Second generation. The blood of the heart of one rabbit that had died of one-hundredth of a drop was inoculated in three rabbits, in doses of $\frac{100}{1000}$, $\frac{1}{1000000}$ and $\frac{10}{1000000}$ of a drop. All died, in intervals of sixteen to twenty-three hours.

"Third generation. The blood of a rabbit that had died of the $\frac{10}{1000}$ of a drop, was injected in five rabbits, in doses $\frac{100}{1000}$, $\frac{1}{1000000000}$ and $\frac{1}{1000000000000}$ of a drop. All died in from twenty-four to twenty-five hours. These facts prove sufficiently that septicæmic virus acquires suddenly its greatest power.

"I will not undertake to search if the virus which acquires so great an activity in the economy of an animal is of another nature than that of the virus of the putrefied substance that had killed this animal. I can say that it is not the putrefaction of the cadaver that has impressed a new activity to the poison found in its veins; for, in my experiments, often the inoculation has been practiced immediately after death, the body being still warm.

"But I will say more: The septicæmic virus very probably is destroyed by the putrefaction which seizes the animal that it has killed. Here are two experiments that respond to this question:

"First experiment. The blood of a rabbit that had died of septicæmia the 28th of July, (the virulence of which had been established by inoculating rabbits,) was preserved in a flask twenty-three days. The 20th of August it was inoculated in three cobayas, in doses of one-eighth, one-fortieth and one-hundredth of a drop. The result was altogether nul.

"Second experiment. The blood of a septicæmic rabbit that died on the 19th of August, making part of a series that died from doses of $\frac{1}{1000000000000}$ of a drop, was preserved in a flask twenty days. The 8th of September, a rabbit was inoculated with the blood in a dose of $\frac{2}{1000}$ of a drop. This rabbit was not even indisposed.

"These facts, if sustained by further experiments and observations, possess more than ordinary interest; they explain also the observations of Dr. Calles, relative to the minimum virulence of cadavers in advanced putrefaction."

